# Neurotics and Cardio-Vascular Neuroses

**Published:** 1898-04-02

**Authors:** 


### NEUROTICS AND CARDIO-VASCULAR NEUROSES.

In the Lumleian Lectures now being delivered before the Royal College of Physicians, Sir R. Douglas Powell deals with the Principles which Govern Treatment in Diseases and Disorders of the Heart. He begins by treating of such modifications of the cardiac functions as are the results of disturbed or even organically changed innervation of the heart and vessels, the heart itself remaining sound, for he takes the very useful position that all such causes are apt to act equally upon hearts that are unsound, and that it is the occurrence in cases of organically diseased hearts of what, when they occur in sound hearts, are spoken of as functional disorders, that causes many of the symptoms of heart disease. Functional disturbance is, he says, as frequently observed in association with diseased as with healthy hearts, and many of the troubles and some of the catastrophes of cardiac disease are attributable to functional derangement. The difference lies in the result. "Numberless people die from functional disturbance of a diseased heart, few or none die from a functional disturbance of a sound heart."

His definition of a cardio-vascular neurosis is as follows: An increased sensibility, and a disordered action of the heart and vessels not dependent upon structural change. There are a large number, perhaps an increasing number, of people who are morbidly conscious of their heart's action, and, indeed, of the function of their cardio-vascular system generally, or in particular parts. They feel disturbances, flutterings at the heart, throbbings, flushings, pallors of the surface or of particular organs, noises in the vessels about the head, and are conscious of the beating of their heart, and of any disorder of, or faintness in, its action. This condition and the thousand and one variations to which it is subject are the result of purely nervous disorder. It is among the class of so-called neurotics that functional disorders of the heart so largely abound; and, if one has to define what is meant by a "neurotic person," one may say that such a person is one whose whole nervous system, and especially that portion of it known as the organic nervous system, is hypersensitive and abnormally within his cognisance. The term, "neurotic," however, must not be looked on as a term of reproach. It is not necessarily connected with hysteria, which indeed is but one of its lesser manifestations. It is a condition of nervous system which is sometimes acquired but more often is hereditary, which is very widely prevalent, and is a factor which has always to be reckoned with in practical medicine; for whilst sometimes found amongst the self-indulgent it is quite as often seen in persons with a high degree of self-control and a restless energy and enterprise, and is especially prevalent among the educated classes. The tendency of the neurotic is to intemperance in thought and action and even in suffering, so that while we may be dazzled by his mental brilliancy, his powers of acquisition, and his rapidity of thought, we may, as physicians, be more often bewildered by his powers of suffering. Just as some persons are gifted with an intensity of hearing which enables them almost painfully to appreciate the overtones which occupy etherial spaces which are dumb and dark to their fellows, so there are persons who are in an exaggerated degree sensible of their own organic mechanism even when acting in only a physiological manner.

There may then be said to be three stages or degrees of cardio-vascular hyperæsthesia. The first, consisting of an undue appreciation of the heart's action and of the blood circulating through the vessels, is extremely common in nervous introspective people, the heart's action being quite normal, but liable from slight causes to become paroxysmally excited and accelerated. These patients are generally of the neurotic class, but alcoholic, social, and tobacco excesses will produce the same effect.

The next degree is that in which the heart's action is really oppressed. Increased arterial tension is an essential factor in this class of cases. These persons may not be introspective; their attention is not concentrated upon their circulation, but is compelled thereto by actual discomfort or pain, and it is interesting to note that that vague and ill-defined anxiety, concentrated about the heart, of which so many of these patients complain, is closely allied in character, and probably in mechanism, but in very mitigated degree, with that cardiac terror so characteristic of angina pectoris, the condition which may be taken as indicating the third stage or degree of cardio-vascular neurosis.

